# On the Statistical Errors of RADAR Location Sensor Networks with Built-In Wi-Fi Gaussian Linear Fingerprints

**DOI:** 10.3390/s120303605

**Published:** 2012-03-15

**Authors:** Mu Zhou, Yu Bin Xu, Lin Ma, Shuo Tian

**Affiliations:** Communication Research Center, School of Electronics and Information Engineering, Harbin Institute of Technology, 2 Yikuang Street, Nangang District, Harbin, Heilongjiang 150080, China; E-Mails: ybxu@hit.edu.cn (Y.B.X.); malin@hit.edu.cn (L.M.); ts0805101@126.com (S.T.)

**Keywords:** RADAR localization, RSS sensing, statistical errors, probabilistic fingerprints, Gaussian distribution

## Abstract

The expected errors of RADAR sensor networks with linear probabilistic location fingerprints inside buildings with varying Wi-Fi Gaussian strength are discussed. As far as we know, the statistical errors of equal and unequal-weighted RADAR networks have been suggested as a better way to evaluate the behavior of different system parameters and the deployment of reference points (RPs). However, up to now, there is still not enough related work on the relations between the statistical errors, system parameters, number and interval of the RPs, let alone calculating the correlated analytical expressions of concern. Therefore, in response to this compelling problem, under a simple linear distribution model, much attention will be paid to the mathematical relations of the linear expected errors, number of neighbors, number and interval of RPs, parameters in logarithmic attenuation model and variations of radio signal strength (RSS) at the test point (TP) with the purpose of constructing more practical and reliable RADAR location sensor networks (RLSNs) and also guaranteeing the accuracy requirements for the location based services in future ubiquitous context-awareness environments. Moreover, the numerical results and some real experimental evaluations of the error theories addressed in this paper will also be presented for our future extended analysis.

## 1. Introduction

The significantly growing interest in ubiquitous computing and context-awareness applications has required reliable, accurate and real-time localization technologies to locate the users’ positions in high-speed, seamless and heterogeneous wireless personal networks (WPNs), especially in the in-building environments where the Global Navigation Satellite System (GNSS) and geolocation in cellular system are not accurate enough [1–3]. Ranging from the military to public uses, from the urban to rural regions, and from the outdoor to indoor areas, location based services (LBSs) were already widely favored and popularized in the recent decade [4–6]. The typical LBSs mainly involve human navigation in unfamiliar buildings, robot path planning and guidance, health care inside modern hospitals, location-based enhanced sensing, entity and storage tracking and management. In GNSS, the Global Positioning System (GPS) can provide 10 m accuracy for the standard positioning service [7]. Global Navigation Satellite System (GLONASS) can achieve 1.5 m accuracy for civilian use [8]. The Galileo Positioning System is supposed to provide the highest 1 m accuracy for civilian applications [9]. The Beidou System is planned to offer the 20 m accuracy with only three satellites [10]. However, the accuracy in all these systems will be seriously deteriorated in closed in-building environments by the infrastructure barriers, body shadowing, RSS attenuation and multi-path interference [11–13].

In the meantime, there is also a large body of in-building localization or navigation systems which can be commonly categorized into fingerprint, model and measurement-based systems [14–16]. With the help of RSS sensing from each visible Wi-Fi access point (AP) or the Wi-Fi wireless router, the world’s first and most representative fingerprint-aided RADAR system was presented by Microsoft Research in 2000 [17]. Cambridge’s Active Bat can find users’ positions by calculating the time difference of arrival (TDOA) between the ultrasound and radio frequency signals based on a multi-lateration algorithm [18]. Carnegie Mellon’s CMU-PM and CMU-TMI location systems, respectively, rely on the Manhattan distance and offset mapping relations of each fingerprint [19]. UCLA’ Nibble system can be suggested as the first signal to noise ratio (SNR)-based localization system in Bayesian networks [20]. The Horus system invented by The University of Maryland can be recognized as a practical and efficient solution to the small-scale attenuation compensation problem with the help of continuous space estimation and location clustering algorithms [21]. MIT’s Cricket system has been used to build interactive video games through the interaction between different pervasive computing devices and has also achieved better performance in location privacy, scalability and tracking agility [22]. In addition, The Pitt’s Voronoi system [23] and RWTH’s hidden Markov localizer [24] have also provided some preliminary analyses of how to improve the accuracy of fingerprint-aided localization.

Among them, the Wi-Fi fingerprint-based localization system outperforms the other two systems because of the following three advantages: (1) RSS variations in real in-building areas cannot be easily characterized by a simple attenuation model due to the changes of directions or angles. Therefore, the construction of effective and reliable model-based localization systems will always involve high labor and time costs [25]; (2) meanwhile, measurement-based systems (e.g., the arriving time, time of difference and angles) will require special infrastructure and deployment which consequently results in higher maintenance and energy consumption [26]; (3) fingerprint-based systems rely on existing lower-priced Wi-Fi devices, non-registered 2.4 GHz ISM band and free 802.11 b/g protocol licenses [27].

The Wi-Fi fingerprint-based localization in RLSNs generally consists of the following three steps [28–30]: (1) in the off-line (or calibration) phase, the Wi-Fi APs are deployed to provide sufficient and seamless RSS coverage in the target location areas, which means, at any physical position, the user can detect and sense the continuous-time RSS from two or more visible APs; (2) the coordinates of pre-calibrated RPs (or the physical locations for fingerprint matching) and their associated RSS samples will be saved as the fingerprints in the radio map. At this point, the fingerprints can be suggested as the mapping relationships between the physical coordinates and pre-sensed RSS values. For example, the fingerprints in RLSNs can be defined as the mapping relations between the 2-D coordinates and user datagram protocol (UDP) RSS samples; (3) in the on-line (or estimation) phase, by matching the new sensed RSS to the pre-stored fingerprints (fingerprint matching), the users’ positions will be estimated by the equal or unequal-weighted sum of the (*K*) neighbors’ coordinates.

Therefore, we can observe that the statistical errors in RLSNs depend significantly on the fingerprint recording in the off-line phase and fingerprint matching in the on-line phase. To the best of our knowledge, three typical models are commonly used for studying the statistical errors in fingerprint-based RLSNs, known respectively as the experimental model, node-pair model and random model. The first model always involves significant labor and time cost, but it can be suggested as the simplest way to evaluate the performance and satisfy the industrial requirements [17]. The second one involves the idea of examining the RSS difference in each RPs’ pair. In this case, the bigger the overlap of the RSS distributions, the larger the statistical errors that will be probably induced [23]. The last one normally relies on computer simulations (e.g., the Monte Carlo method) with lower practical similarities [31].

This paper is divided as follows: Section 2 provides an overview of the in-building RADAR system in RLSNs and some related work on the statistical errors. In Section 3, with a general idea of the simple linear distribution model, the mathematical relations about the expected linear errors in the RLSNs are significantly discussed using the assumption of a logarithmic Gaussian strength-varying model. In Section 4, some numerical and experimental results in the equal and unequal-weighted RLSNs are addressed. Finally, the conclusions and challenges for our future extended work are summarized in Section 5.

## 2. Related Work

### 2.1. Architecture of RADAR System in RLSNs

**Figure 1.**
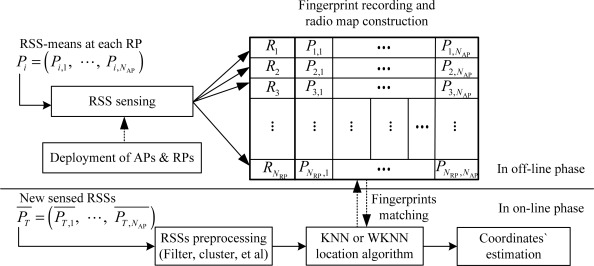
Fingerprint-based RADAR localization system in Wi-Fi RLSNs.

As we know, the fingerprint-based RADAR system in Wi-Fi RLSNs is also called the *K* nearest neighbors (KNN) or weighted *K* nearest neighbors (WKNN) localization, shown in Figure 1. Moreover, RADAR localization system can be recognized as a global matching process between the new sensed RSS and pre-stored fingerprints in a radio map, and find the front *K* RPs with smaller RSS difference for the coordinates’ estimation. However, by the KNN or WKNN location algorithm, although the pre-sensed RSS-mean at the RPs can be normally characterized by some distance dependence models, the on-line new recorded RSS will always vary a lot. Therefore, if we assume the Gaussian model satisfied at the TP, the larger standard deviations will consequently result in larger confidence probabilities of selecting the physically distant RPs as the neighbors.

From Figure 1, we can observe that although the radio map construction involves a high labor cost and cumbersome work for the deployment of RPs and associated RSS sensing, it should be done during the off-line phase. Further, the fingerprint matching by KNN and WKNN algorithms will also significantly influence the accuracy performance of the RADAR system in RLSNs. The estimated positions $C_{\text{KNN}}^{*}$ and $C_{\text{WKNN}}^{*}$ by the KNN and WKNN algorithms are calculated respectively in Equation (1).

(1) $$\left\{ \begin{array}{l} \rho =\left\{\rho \left(R_{i}\right)\right\},\;\rho \left(R_{i}\right)={\left(\sum_{t=1}^{N_{\text{AP}}}{\left|\bar{P_{T,t}}-P_{i,t}\right|}^{2}\right)}^{1/2},\;\\ \gamma_{K}=\left\{R_{j}:\;\rho \left(R_{j}\right)\in \lambda_{K}\;\left(\rho \right),\;j=1,\;\cdots ,\;K\right\},\\ \lambda_{K}\;\left(\rho \right)=\left\{\rho \left(R_{s}\right):\;\rho \left(R_{s}\right)\leq \text{min}\left\{\rho \backslash \lambda_{K}\;\left(\rho \right)\right\}\right\},\;N\left\{\lambda_{K}\;\left(\rho \right)\right\}=K,\\ C_{\text{KNN}}^{*}=\sum_{j=1}^{K}R_{j}/K=\left(\sum_{j=1}^{K}x_{j}/K,\;\sum_{j=1}^{K}y_{j}/K\right),\\ C_{\text{WKNN}}^{*}=\sum_{j=1}^{K}\bar{w_{j}}R_{j}=\left(\sum_{j=1}^{K}\bar{w_{j}}x_{j},\;\sum_{j=1}^{K}\bar{w_{j}}y_{j}\right),\\ R_{j}=\left(x_{j},\;y_{j}\right)\in \gamma_{K}\subset \left\{R_{i}:\;i=1,\;\cdots ,\;N_{\text{RP}}\right\},\;\bar{w_{j}}=p_{j}/\sum_{z=1}^{K}p_{z},\\ \bar{P_{T}}=\left(\bar{P_{T,1}},\;\cdots ,\;\bar{P_{T,N_{\text{AP}}}}\right);\;P_{i}=\left(P_{i,1},\;\cdots ,\;P_{i,N_{\text{AP}}}\right) \end{array}\right.$$

where *N*_AP_ and *N*_RP_ are the numbers of APs and RPs; $\bar{P_{T}}$ and *P_i_* respectively denote the expectation of the RSS at TP and RSS-mean at the RP; *R_i_*; *N*{λ*_K_*(***ρ***)} is the number of elements of the set λ*_K_*(***ρ***); *p_z_*(*z* = 1, ⋯ ,*K*) represents the probability of *R_z_* to be selected as the most RSS-adjacent RP.

### 2.2. Previous Work on Statistical Errors of RADAR System in RLSNs

As mentioned before, there are three types of modeling for evaluating the localization errors in the RSS-based RLSNs as follows:

1. Experimental model. This model is the simplest one for wide industrial applications and overall system performance evaluation. However, during the modeling, we need to experimentally discuss the performance of each technical parameter under different fingerprint conditions to find the best system architecture. Therefore, this model will consequently involve of a large amount of labor and time costs in the off-line phase [12,17,21] and [29].
2. Node-pair model. In this model, we always assume the Wi-Fi APs are located symmetrically in the location area, RPs are calibrated uniformly as grids, and the logarithmic Gaussian attenuation channel is satisfied. The most representative work about this model can be found in [23]. Although there are some preliminary analytical results addressed in that paper that can be applied to 2D areas, the accuracy in RLSNs cannot be effectively guaranteed when the number of neighbors is larger than 1 because this model mainly focuses on the RSS relations in each pair of RPs. Meanwhile, the overlapping of the RSS distributions in Gaussian model is suggested as the reason for localization errors. At this point, if we make an assumption there are two RPs, *R_s_* and *R_t_* respectively with RSS-mean *P_s_* = (*P*_s,1_, ⋯ ,*P*_*s,N*_AP__) and *P_t_* = (*P*_t,1_, ⋯ ,*P*_*t,N*_AP__), and *R_s_* is addressed as our target matching (or the correct matching with the smallest RSS distance to the new sensed RSS), then, the confidence probability of the matching equals to $\text{Prob}\{|P_{s}-\bar{P_{T}}|\;\leq \;|P_{t}-\bar{P_{T}}|\}=\int_{-\infty }^{0}\frac{1}{\sqrt{2\pi }\sigma_{\lambda }}e^{\frac{{\left(\lambda -\mu_{\lambda }\right)}^{2}}{{2\sigma }_{\lambda }^{2}}}\text{dz}\;=1-Q\left(-\frac{\mu_{\lambda }}{\sigma_{\lambda }}\right)$, where, $\lambda =2\sum_{j=1}^{N_{\text{AP}}}\bar{P_{T,J}}\left(P_{t,j}-P_{s,j}\right)+\sum_{j=1}^{N_{\text{AP}}}\left(P_{s,j}^{2}-P_{t,j}^{2}\right)$; $\mu_{\lambda }=2\sum_{j=1}^{N_{\text{AP}}}P_{S,j}\left(P_{t,j}-P_{s,j}\right)+\sum_{j=1}^{N_{\text{AP}}}\left(P_{s,j}^{2}-P_{t,j}^{2}\right)$ and $\sigma_{\lambda }^{2}=4\sum_{j=1}^{N_{\text{AP}}}{\left(P_{t,j}-P_{s,j}\right)}^{2}\sigma_{s,j}^{2}$ ; *σ_s,j_* denotes the standard deviation of pre-sensed RSS from the *j*-th AP at *R_s_*.
3. Random model. Under this model, the RPs are assumed to be located randomly (or distributed by the Monte Carlo simulations), the parameter “physical distance to each AP” in logarithmic channel will be normally simplified to some other special measurements [31] (e.g., the log-attenuation property is supposed to be satisfied in every direction from each RP, not just in the direction “from AP to RP”).

Overall, as a general and simple model of the in-building straight corridors, the analytical analysis on the statistical errors in linearly distributed RPs environment seems to be much more necessary and important than the other environments, such as the offices, washrooms and meeting rooms. Further, the differences with the previous work about statistical error discussions can generally be summarized as follows: (1) A better RADAR system in RLSNs with higher expected localization accuracy can be designed only with the help of the analytical relations addressed in the paper, but not involving any cumbersome work for the experimental evaluations. Therefore, the labor and time costs can be saved; (2) There are *K* (*K* > 2) neighboring RPs to be considered together in the on-line estimation phase, not just one pair of two RPs. Meanwhile, the linear model introduced in this paper can also be verified to perform much more effectively because the increase of *K* will also improve the localization accuracy; (3) Last but not least, the deployment of RPs with distance interval *r* is more practical and reasonable compared to the random models and it will also achieve lower cost for the fingerprint recording.

As far as we know, the RSS-based in-building localization in RLSNs has been widely favored in applications ranging from the military to public uses. For example, for both travelers and robots in unfamiliar environments, it is necessary to locate their positions in real-time and provide navigation services or ease the complexity of path planning. Compared to the traditional ultra-sound and LADAR location sensor networks in the in-building areas, the Wi-Fi fingerprint-based RLSNs will provide a better alternative way to locate the users in the aspects of infrastructure cost and accuracy performance. Currently, in lots of modern hospitals, schools and health care centers, the elderly, disabled people or children will always need to be located or tracked by their doctors or parents. If there is an emergency or someone is out of his/her permitted area, the doctors or parents will be notified in real-time and acknowledged with the help of the interaction between the service centers, APs and Wi-Fi RSS sensors attached on the people’s body.

### 2.3. Notations and Parameters in RLSNs

In the results that follow, the notations and parameters used are listed in Table 1.

## 3. Mathematical Expressions of Expected Linear Errors in RLSNs

### 3.1. General Idea of a Simple Linear Distribution Model

**Figure 2.**
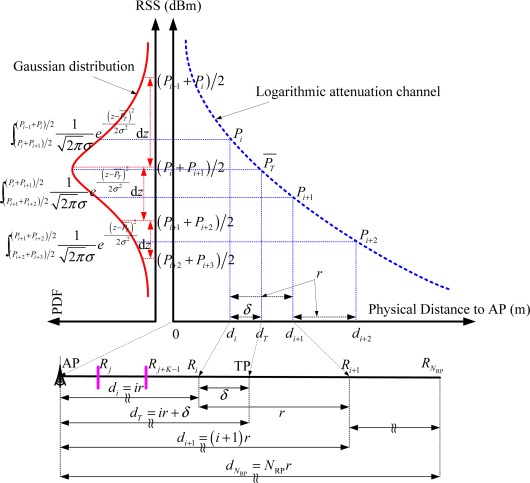
Linear distribution model in logarithmic Gaussian variation channel.

As shown in Figure 2, there are *N*_RP_ RPs uniformly calibrated in the linear location area with distance *d_i_* = *ir* to the AP. The pre-sensed RSS-mean at each RP and the new sensed RSS-mean at TP are respectively calculated by the logarithmic channel *P_i_* = *P_t_* − *L*_0_ − 10*α*lg*d_i_* and *P_T_* = *P_t_* − *L*_0_ − 10*α*lg*d_T_*. The geometrical relations and associated RSS distributions to be discussed are also presented in Figure 2. By the assumption of Gaussian RSS variations at TP, the confidence probability of *R_i_* to be selected as the most RSS-adjacent neighbor is calculated by the integral $\int_{\frac{\left(P_{i}+P_{i+1}\right)}{2}}^{\frac{\left(P_{i-1}+P_{i}\right)}{2}}\frac{1}{\sqrt{2\pi }\sigma }e^{-\frac{{\left(z-\bar{P_{T}}\right)}^{2}}{{2\sigma }^{2}}}dz$.

As shown in Figure 2, by the assumptions of logarithmic Gaussian attenuation channel and continuous RSS variations, the statistical error evaluations in our linear distribution model mainly consist of the following three steps: (1) In the linearly distributed RPs condition, the neighbor set that consists of the neighboring RPs with smaller RSS distance to the new sensed RSS is denoted by {*R_j_*, ⋯ ,*R*_*j*+*K*−1_}. Meanwhile, the confidence probability of this set ${\text{Prob}}_{K}^{j}(j=1,\cdots ,N_{\text{RP}}-K+1)$ depends significantly on the Gaussian RSS variations at TP. Further, in this paper, there are three cases for different values of *j* conditions that need to be discussed; (2) From Equation (1), we will calculate $C_{\text{KNN}}^{*}$ and $C_{\text{WKNN}}^{*}$ in equal and unequal-weighted RLSNs, respectively, by the KNN and WKNN algorithms with neighbor set {*R_j_*, ⋯ ,*R_j_*_+_*_K_*_−1_}; (3) Finally, the statistical errors in the equal and unequal-weighted RLSNs (*ε_K_* and *ε_WK_*) will be calculated, respectively, by the expectations of error functions $\varepsilon_{K}=\sum_{j=1}^{N_{\text{RP}}-K+1}{\text{Prob}}_{K}^{j}C_{\text{KNN}}^{*}$ and $\varepsilon_{\mathrm{WK}}=\sum_{j=1}^{N_{\text{RP}}-K+1}{\text{Prob}}_{K}^{j}C_{\text{WKNN}}^{*}$ with respect to the random variables *i*, *j* and *δ*.

### 3.2. Errors in Equal-Weighted RLSNs

In the first step, we need to discuss the following three cases in different neighbor sets conditions, respectively. Based on the Gaussian variations of the new sensed RSS at TP, the probability of each case will significantly depend on the values of *j* (*j* = 1, *j* = 2, ⋯ ,*N*_RP_ − *K* and *N*_RP_ − *K +* 1).

**Figure 3.**
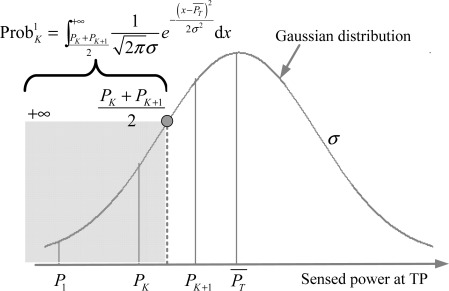
Confidence probability of case 1 in equal-weighted RLSNs.

**Figure 4.**
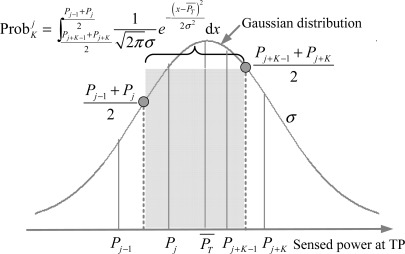
Confidence probability of case 2 in equal-weighted RLSNs.

**Figure 5.**
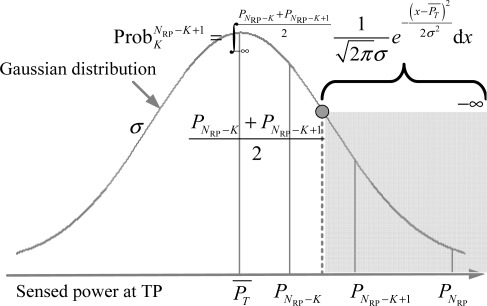
Confidence probability of Case 3 in equal-weighted RLSNs.

1. Case 1: *j* = 1 with the neighbor set {*R*_1_, ⋯ ,*R_K_*}. In this case, because there is no RP between the AP and *R*_1_, the new sensed RSS at TP should fall in the range of $[\frac{P_{K}+P_{K+1}}{2},+\infty )$, as shown in Figure 3. The confidence probability of set {*R*_1_, ⋯ ,*R_K_*} is calculated by Equation (2), where, $Q(x)=\int_{x}^{+\infty }\frac{1}{\sqrt{2\pi }}e^{-\frac{z^{2}}{2}}dz$ and (*x*) + *Q* (−*x*) = 1.
  (2) $${\text{Prob}}_{K}^{1}=\int_{\frac{P_{K}+P_{K+1}}{2}}^{+\infty }\frac{1}{\sqrt{2\pi }\sigma }e^{-\frac{{\left(x-\bar{P_{T}}\right)}^{2}}{{2\sigma }^{2}}}dx=Q\left(\frac{\alpha }{\sigma }\text{lg}\frac{\mathrm{ir}+\delta }{r\sqrt{K\left(K+1\right)}}\right)$$
2. Case 2: *j* = 2, ⋯ ,*N*_RP_ − *K* with the neighbor set {*R_j_*, ⋯ ,*R_j+K_*_−1_}. As shown in Figure 4, the confidence probability in this case equals to the cumulative probability from $\frac{P_{j+K-1}+P_{j+K}}{2}$ to $\frac{P_{j-1}+P_{j}}{2}$ (see Equation (3)) by the Gaussian RSS variations.
  (3) $${\text{Prob}}_{K}^{j}=\int_{\frac{P_{j+K-1}+P_{j+K}}{2}}^{\frac{P_{j-1}+P_{j}}{2}}\frac{1}{\sqrt{2\pi }\sigma }e^{-\frac{{\left(x-\bar{P_{T}}\right)}^{2}}{{2\sigma }^{2}}}dx=Q\left(\frac{\alpha }{\sigma }\text{lg}\frac{\mathrm{ir}+\delta }{r\sqrt{\left(j+K-1\right)\left(j+K\right)}}\right)-Q\left(\frac{\alpha }{\sigma }\text{lg}\frac{\mathrm{ir}+\delta }{r\sqrt{\left(j-1\right)j}}\right)$$
3. Case 3: *j* = *N*_RP_ − *K* + 1 with the neighbor set {*R*_*N*_RP_− *K*+1_, ⋯ ,*R*_*N*_RP__}. Similar to the analysis in case 1, the confidence probability in this case (see Equation (4)) should be calculated by the integral from the minus infinity to $\frac{P_{N_{\text{RP}}-K}+P_{N_{\text{RP}}-K+1}}{2}$, as shown in Figure 5.
  (4) $${\text{Prob}}_{K}^{N_{\text{RP}}-K+1}=\int_{-\infty }^{\frac{P_{N_{\text{RP}}-K}+P_{N_{\text{RP}}-K+1}}{2}}\frac{1}{\sqrt{2\pi }\sigma }e^{-\frac{{\left(x-\bar{P_{T}}\right)}^{2}}{{2\sigma }^{2}}}dx=Q\left(\frac{\alpha }{\sigma }\text{lg}\frac{r\sqrt{\left(N_{\text{RP}}-K\right)\left(N_{\text{RP}}-K+1\right)}}{\mathrm{ir}+\delta }\right)$$

In the second step, when the RPs *R_j_*, ⋯ ,*R_j_*_+_*_K_*_−1_(*j* = 1, ⋯ ,*N*_RP_ − *K* + 1) are selected as neighbor set {*R_j_*, ⋯ ,*R_j_*_+_*_K_*_−1_}, the errors between the real position of TP and $C_{\text{WKNN}}^{*}\;(\varepsilon_{K}^{j})$ in the equal-weighted RLSNs are calculated by Equation (5).

(5) $$\varepsilon_{K}^{j}=\left|\text{TP}-C_{\text{KNN}}^{*}\right|=\left|\left(\mathrm{ir}+\delta \right)-\frac{\left(2j+K-1\right)r}{2}\right|=\left|\left(i-j\right)r+\left(\delta -\frac{\left(K-1\right)r}{2}\right)\right|$$

Finally, in the last step, the linear expected errors in the equal-weighted RLSNs equals to:

(6) $$\begin{array}{l} \varepsilon_{K}=E_{i}E_{j}E_{\delta }\;\left\{\sum_{j=1}^{N_{\text{RP}}-K+1}{\text{Prob}}_{K}^{j}\varepsilon_{K}^{j}\right\}=E_{i}E_{\delta }\left\{\frac{{\text{Prob}}_{K}^{1}\varepsilon_{K}^{1}+\sum_{j=2}^{N_{\text{RP}}-K}{\text{Prob}}_{K}^{j}\varepsilon_{K}^{j}+{\text{Prob}}_{K}^{N_{\text{RP}}-K+1}\varepsilon_{K}^{N_{\text{RP}}-K+1}}{\sum_{\lambda =1}^{N_{\text{RP}}-K+1}{\text{Prob}}_{K}^{\lambda }}\right\}\\ =\frac{1}{N_{\text{RP}}}\sum_{i=1}^{N_{\text{RP}}}\left(\frac{{\text{Prob}}^{1}\varepsilon^{1}+\sum_{j=2}^{N_{\text{RP}}-K}{\text{Prob}}^{j}\varepsilon^{j}+{\text{Prob}}^{N_{\text{RP}}-K+1}\varepsilon^{N_{\text{RP}}-K+1}}{\sum_{\lambda =1}^{N_{\text{RP}}-K+1}{\text{Prob}}^{\lambda }}\right),\;{\text{Prob}}^{\lambda }={{\text{Prob}}_{K}^{\lambda }|}_{\delta =r/2},\;\varepsilon^{\lambda }={\varepsilon_{K}^{\lambda }|}_{\delta =r/2}. \end{array}$$

where, ${\text{Prob}}_{K}^{\lambda }{|}_{\delta =r/2}$ and $\varepsilon^{\lambda }=\varepsilon_{K}^{\lambda }{|}_{\delta =r/2}$ respectively stand for the values of ${\text{Prob}}_{K}^{\lambda }$ and $\varepsilon_{K}^{\lambda }$ when *δ* = *r*/2.

### 3.3. Errors in Unequal-Weighted RLSNs

From Equation (1), the difference between the equal and unequal-weighted RLSNs is about the weights’ distribution in the neighbor set. The estimated position in equal-weighted RLSNs $C_{\text{KNN}}^{*}$ is located at the geometric center of the neighbor set {*R_j_*, ⋯ ,*R_j_*_+_*_K_*_−1_} because the equal weight 1/*K* is distributed to each neighbor. However, in the unequal-weighted RLSNs, the weight of each neighbor significantly relies on the confidence probability to be selected as the most RSS-adjacent RP ${\text{prob}}_{\mathrm{WK}}^{s}$ (for RP *R_s_*). Therefore, we will also need to discuss the following three cases in different values of *j* conditions:

1. Case 1: *j* =1 with the neighbor set {*R*_1_, ⋯ ,*R_K_*}. In this case, the confidence probability of *R_l_*{*l* = 1, ⋯ ,*K*} to be selected as the most RSS-adjacent RP ${\text{prob}}_{\mathrm{WK}}^{l}$ can be calculated by Equation (7).
  (7) $${\text{prob}}_{\mathrm{WK}}^{\ell }=\left\{ \begin{array}{l} \int_{\frac{P_{1}+P_{2}}{2}}^{+\infty }\frac{1}{\sqrt{2\pi }\sigma }e^{-\frac{{\left(x-\bar{P_{T}}\right)}^{2}}{{2\sigma }^{2}}}dx=Q\left(\frac{\alpha }{\sigma }\text{lg}\frac{\mathrm{ir}+\delta }{\sqrt{2}r}\right),\;\text{when}\;\ell =1\\ \int_{\frac{P_{\ell }+P_{\ell +1}}{2}}^{\frac{P_{\ell -1}+P_{\ell }}{2}}\frac{1}{\sqrt{2\pi }\sigma }e^{-\frac{{\left(x-\bar{P_{T}}\right)}^{2}}{{2\sigma }^{2}}}dx=Q\left(\frac{\alpha }{\sigma }\text{lg}\frac{\mathrm{ir}+\delta }{r\sqrt{\ell \left(\ell +1\right)}}\right)-Q\left(\frac{\alpha }{\sigma }\text{lg}\frac{\mathrm{ir}+\delta }{r\sqrt{\left(\ell -1\right)\ell }}\right),\;\text{when}\;2\leq \ell \leq K \end{array}\right.$$
  Therefore, the errors between the real and estimated position of TP $\varepsilon_{\mathrm{WK}}^{1}$ in this case equal to:
  (8) $$\varepsilon_{\mathrm{WK}}^{1}=\left|\text{TP}-C_{\text{WKNN}}^{*}\right|=\left|d_{T}-\frac{\sum_{\ell =1}^{K}{\text{prob}}_{\mathrm{WK}}^{\ell }d_{\ell }}{\sum_{\ell =1}^{K}{\text{prob}}_{\mathrm{WK}}^{\ell }}\right|=\left|\left(\mathrm{ir}+\delta \right)-\frac{r\sum_{\ell =1}^{K}\ell {\text{Prob}}_{\mathrm{WK}}^{\ell }}{{\text{Prob}}_{K}^{1}}\right|$$
2. Case 2: *j* = 2, ⋯ ,*N*_RP_ − *K* with the neighbor set {*R_j_*, ⋯ ,*R_j_*_+_*_K_*_−1_}. Similarly, the confidence probability of selecting *R*_*j*+*m*_{*m* = 0, ⋯ ,*K* − 1} as the most RSS-adjacent RP ${\text{prob}}_{\mathrm{WK}}^{j+m}$ and the associated errors $\varepsilon_{\mathrm{WK}}^{j}$ are respectively calculated by Equations (9) and (10):
  (9) $${\text{prob}}_{\mathrm{WK}}^{j+m}=\int_{\frac{P_{j+m}+P_{j+m+1}}{2}}^{\frac{P_{j+m-1}+P_{j+m}}{2}}\frac{1}{\sqrt{2\pi }\sigma }e^{-\frac{{\left(x-\bar{P_{T}}\right)}^{2}}{{2\sigma }^{2}}}dx=Q\left(\frac{\alpha }{\sigma }\text{lg}\frac{\mathrm{ir}+\delta }{r\sqrt{\left(j+m\right)\left(j+m+1\right)}}\right)-Q\left(\frac{\alpha }{\sigma }\text{lg}\frac{\mathrm{ir}+\delta }{r\sqrt{\left(j+m-1\right)\left(j+m\right)}}\right)$$
  (10) $$\varepsilon_{\mathrm{WK}}^{j}=\left|\text{TP}-C_{\text{WKNN}}^{*}\right|=\left|d_{T}-\frac{\sum_{m=0}^{K-1}{\text{prob}}_{\mathrm{WK}}^{j+m}d_{j+m}}{\sum_{m=0}^{K-1}{\text{prob}}_{\mathrm{WK}}^{j+m}}\right|=\left|\left(\mathrm{ir}+\delta \right)-\frac{r\sum_{m=0}^{K-1}\left(j+m\right){\text{Prob}}_{\mathrm{WK}}^{j+m}}{{\text{Prob}}_{K}^{j}}\right|$$
3. Case 3: *j* = *N*_RP_ − *K* + 1 with the neighbor set {*R*_*N*_RP_−*K*+1_, ⋯ ,*R*_*N*_RP__}. In this case, ${\text{prob}}_{\mathrm{WK}}^{n}n=N_{\text{RP}}-K+1,\cdots ,N_{\text{RP}}$ and $\varepsilon_{\mathrm{WK}}^{N_{\text{RP}}-K+1}$ can be obtained by Equations (11) and (12):
  (11) $${\text{prob}}_{\mathrm{WK}}^{n}=\left\{ \begin{array}{l} Q\left(\frac{\alpha }{\sigma }\text{lg}\frac{\mathrm{ir}+\delta }{r\sqrt{n\left(n+1\right)}}\right)-Q\left(\frac{\alpha }{\sigma }\text{lg}\frac{\mathrm{ir}+\delta }{r\sqrt{\left(n-1\right)n}}\right),\;\text{when}\;N_{\text{RP}}-K+1\leq n\leq N_{\text{RP}}-1\\ Q\left(\frac{\alpha }{\sigma }\text{lg}\frac{r\sqrt{\left(N_{\text{RP}}-1\right)N_{\text{RP}}}}{\mathrm{ir}+\delta }\right),\text{when}\;n=N_{\text{RP}} \end{array}\right.$$
  (12) $$\varepsilon_{\mathrm{WK}}^{N_{\text{RP}}-K+1}=\left|\text{TP}-C_{\text{WKNN}}^{*}\right|=\left|d_{T}-\frac{\sum_{n=N_{\text{RP}}-K+1}^{N_{\text{RP}}}{\text{prob}}_{\mathrm{WK}}^{n}d_{n}}{\sum_{n=1}^{K}{\text{prob}}_{\mathrm{WK}}^{n}}\right|=\left|\left(\mathrm{ir}+\delta \right)-\frac{r\sum_{n=N_{\text{RP}}-K+1}^{N_{\text{RP}}}n{\text{prob}}_{\mathrm{WK}}^{n}}{{\text{Prob}}_{K}^{N_{\text{RP}}-K+1}}\right|$$

Finally, based on Equations (2–4) and Equations (7–12), the linear expected errors in the unequal-weighted RLSNs will be calculated by Equation (13):

(13) $$\begin{array}{l} \varepsilon_{\mathrm{WK}}=E_{i}E_{j}E_{\delta }\;\left\{\sum_{j=1}^{N_{\text{RP}}-K+1}{\text{Prob}}_{K}^{j}\varepsilon_{\mathrm{WK}}^{j}\right\}=E_{i}E_{\delta }\left\{\frac{{\text{Prob}}_{K}^{1}\varepsilon_{\mathrm{WK}}^{1}+\sum_{j=2}^{N_{\text{RP}}-K}{\text{Prob}}_{K}^{j}\varepsilon_{\mathrm{WK}}^{j}+{\text{Prob}}_{K}^{N_{\text{RP}}-K+1}\varepsilon_{WK}^{N_{\text{RP}}-K+1}}{\sum_{\lambda =1}^{N_{\text{RP}}-K+1}{\text{Prob}}_{K}^{\lambda }}\right\}\\ =\frac{1}{N_{\text{RP}}}\sum_{i=1}^{N_{\text{RP}}}\left(\frac{{\text{Prob}}^{1}\varepsilon_{W}^{1}+\sum_{j=2}^{N_{\text{RP}}-K}{\text{Prob}}^{j}\varepsilon_{W}^{j}+{\text{Prob}}^{N_{\text{RP}}-K+1}\varepsilon_{W}^{N_{\text{RP}}-K+1}}{\sum_{\lambda =1}^{N_{\text{RP}}-K+1}{\text{Prob}}^{\lambda }}\right),\;\varepsilon_{W}^{\lambda }={\varepsilon_{\mathrm{WK}}^{\lambda }|}_{\delta =r/2}. \end{array}$$

where, $\varepsilon_{W}^{\lambda }=\varepsilon_{\mathrm{WK}}^{\lambda }{|}_{\delta =r/2}$ stands for the value of $\varepsilon_{\mathrm{WK}}^{\lambda }$ when *δ* = *r*/2.

## 4. Numerical and Experimental Results

From the analytical discussions in the previous section, we can find that the linear expected errors *ε_K_* and *ε_WK_* in equal and unequal-weighted RLSNs rely significantly on the parameters *K*, *σ* and *N*_RP_. Therefore, in this section, we will firstly present some numerical results about the relations addressed in Section 3, and then, the real experimental evaluations will also be discussed to verify the results of interest through this paper. In the numerical results that follow, we let *α* = 2.

### 4.1. Error Performance with Variations of K

**Figure 6.**
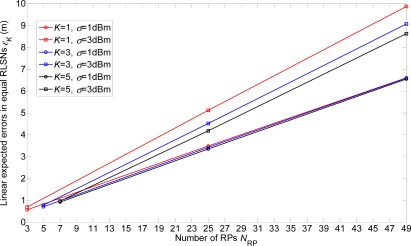
Linear expected errors with variations of *K* in equal-weighted RLSNs (*r* = 0.5 m).

**Figure 7.**
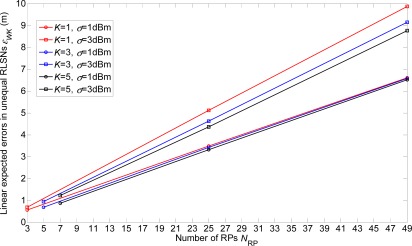
Linear expected errors with variations of *K* in unequal-weighted RLSNs (*r* = 0.5 m).

In Figures 6 and 7, we show the linear expected errors of the equal and unequal-weighted RLSNs, respectively, given that the TP is actually located with the physical distance *d_T_* = *ir* + *δ* to the AP. These are derived from Equations (6) and (13) and plotted as the functions of the number of RPs *N*_RP_. Clearly, as *N*_RP_ increases, the linear expected errors will also increase. However, the errors will not vary a lot with the increase of *K*. Take equal RLSNs for example. At *N*_RP_ = 25 and *σ* = 3 dBm, the decreasing rates of errors from *K* = 1 to *K* = 3, and from *K* = 3 to *K* = 5 are 11.7% and 7.5%.

### 4.2. Error Performance with Variations of r and σ

**Figure 8.**
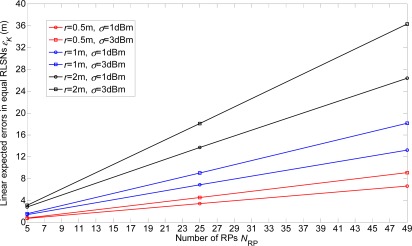
Linear expected errors with variations of *r* and *σ* in equal-weighted RLSNs (*K* = 3).

**Figure 9.**
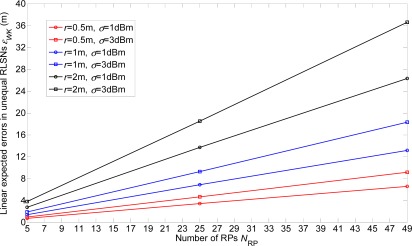
Linear expected errors with variations of *r* and *σ* in unequal-weighted RLSNs (*K* = 3).

**Figure 10.**
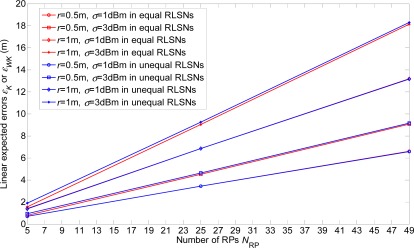
Error comparisons of linear expected errors in equal and unequal-weighted RLSNs.

In this section, the linear expected errors in the equal and unequal-weighted RLSNs are respectively plotted as the functions of *N*_RP_ for various conditions with *r* = 0.5 m, 1 m, 2 m and *σ =* 1 dBm, 3 dBm in Figures 8 and 9. The condition of *r* = 2 m and *σ =* 3 dBm has the largest error, and there are always smaller errors with smaller *r* and *σ* as expected. Furthermore, based on the Figures 6–9, we can observe the influence degree on the linear expected errors in the equal and unequal-weighted RLSNs should be *r* > *σ* > *K*. Therefore, in the following results (in Figures (8–10)), we fix the value of *K* = 3.

### 4.3. Error Comparisons of Equal and Unequal-Weighted RLSNs

The error comparisons between the equal and unequal-weighted RLSNs are shown in Figure 10. It can be easily observed that the errors in equal-weighted RLSNs are slightly smaller the unequal-weighted ones, which can be suggested as another interesting observation from this paper. However, this result can be interpreted based on the following two reasons:

1. In the linearly distributed RPs with logarithmic Gaussian RSS variations, the RSS difference of the distance-adjacent RPs will be significantly diminished as the distance to the AP increases. Then, it cannot be easily guaranteed that the larger weights will be distributed to the distance closer RPs.
2. Because of the significant large confidence probabilities (or infinite integrals) distributed to the closest and farthest RPs *R*_1_ and *R*_*N*_RP__, large errors will be induced in unequal-weighted RLSNs, especially in the small *N*_RP_ and large *σ* conditions.

### 4.4. Experimental Setup

**Figure 11.**
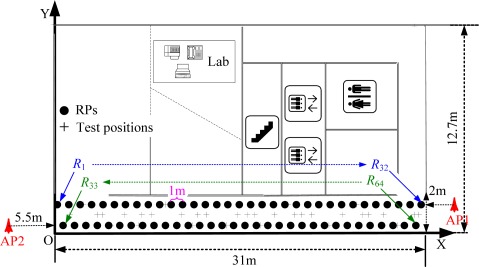
Experimental setup with two APs, 64 RPs and 33 test positions.

In this section, some realistic experimental results about the localization errors in Wi-Fi RLSNs will be carried out in a typical straight corridor environment, as shown in Figure 11. The dimensions of this area are 31 m × 2 m and the RPs are linearly distributed along the corridor with the same 1 m interval.

**Figure 12.**
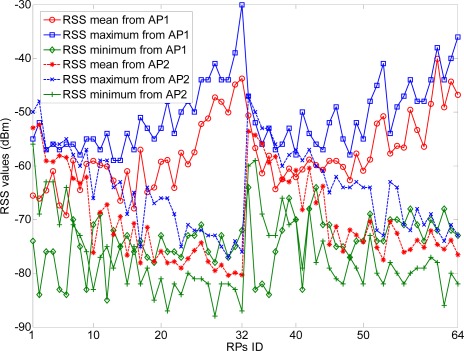
RSS variations in linear distribution model with two LOS visible APs.

There are two line-of-sight (LOS) visible APs (Linksys WAP54G) located at the right and left ends of the corridor at 2 m height, respectively. At each RP (indicated with •’s), there are 300 continuous-time RSS samples recorded for the construction of radio map. The variations of RSS-mean, maximum and minimum are shown in Figure 12. Obviously, if we record the RSS-mean and associated coordinates as the fingerprints, the pre-assumed logarithmic Gaussian channel can be effectively satisfied.

**Figure 13.**
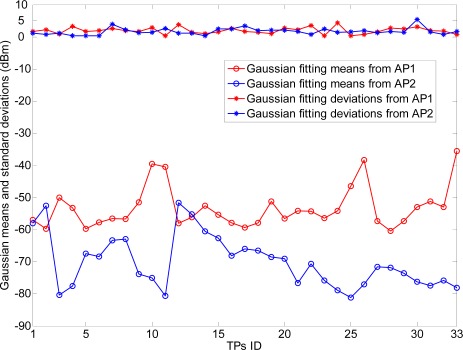
Optimal means and standard deviations in Gaussian fitting models at TPs.

**Figure 14.**
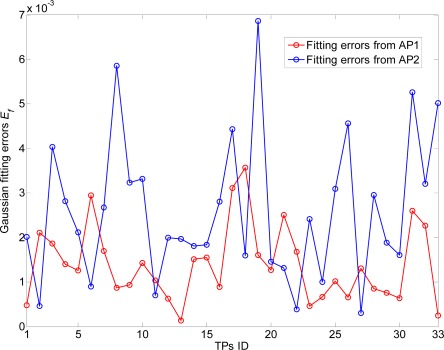
Gaussian fitting errors at TPs.

**Figure 15.**
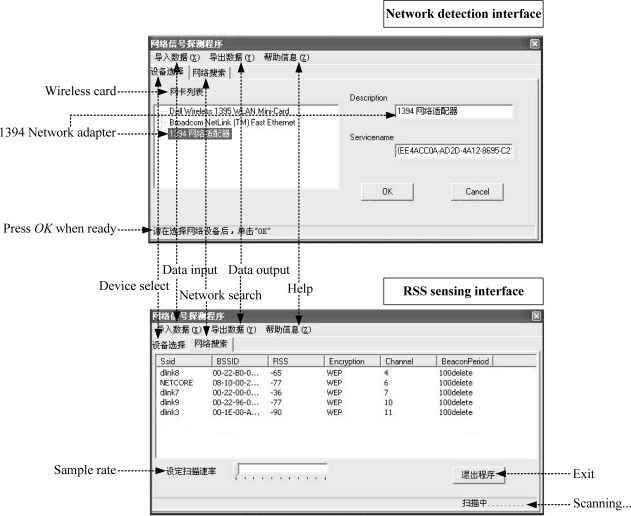
“HITWLAN v1.0” RSS sensing software.

For consistency to our previous mathematical model, the positions of TP (with +’s) are randomly selected in this straight corridor environment. As shown in Figures 13 and 14, the RSS distributions at the TPs (100 new recorded RSS samples per TP) are fitted well by the Gaussian models with small fitting errors $E_{f}=E\{{\left[\text{Prob}(\bar{P_{T}})-\text{Prob}(\bar{P_{T}})\right]}^{2}\}$, where, $\text{Prob}(\bar{P_{T}})$ and $\text{Prob}(\bar{P_{T}})$ denote the real recorded and fitted probabilities of sample $\bar{P_{T}}$. *E_f_* is calculated by the expectation of the probability difference of $\text{Prob}(\bar{P_{T}})$ and $\text{Prob}(\bar{P_{T}})$. Moreover, the RSS samples are recorded by a laptop (ASUS A8F) with our developed RSS sensing software “HITWLAN v1.0” [32] in Figure 15.

### 4.5. Experimental Evaluations in Real In-Building Environments

In this section, we will pay significant attention to the following two observations: (1) The error variations with the increase of *K*, *σ* and *r* in the RLSNs; (2) The error comparisons of equal and unequal-weighted RLSNs.

**Figure 16.**
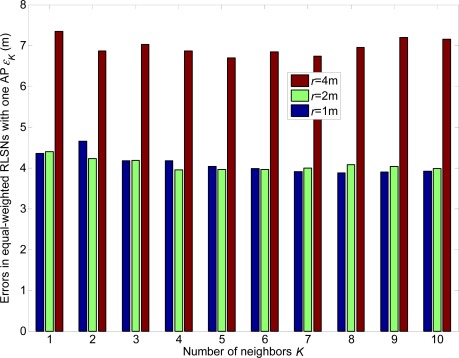
Localization errors in equal-weighted RLSNs with one AP (AP1).

**Figure 17.**
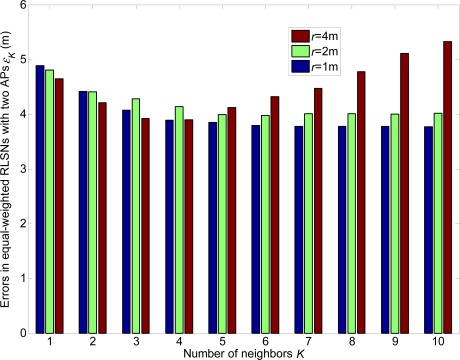
Localization errors in equal-weighted RLSNs with two APs (AP1 and AP2).

**Figure 18.**
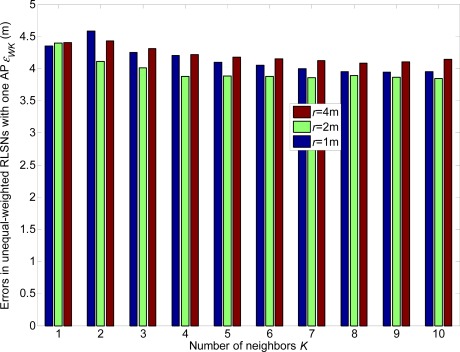
Localization errors in unequal-weighted RLSNs with one AP (AP1).

**Figure 19.**
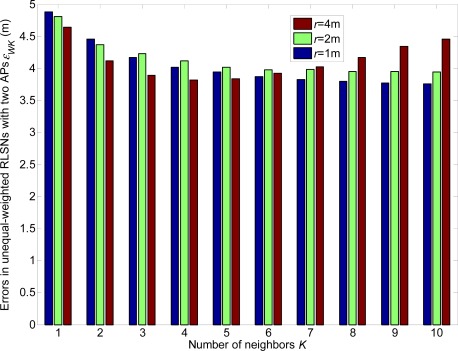
Localization errors in unequal-weighted RLSNs with two APs (AP1 and AP2).

**Figure 20.**
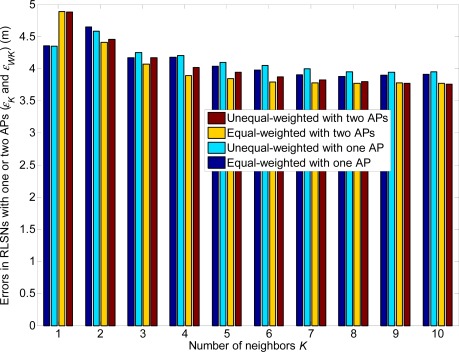
Error comparisons of equal and unequal-weighted RLSNs (*r* = 1 m).

**Figure 21.**
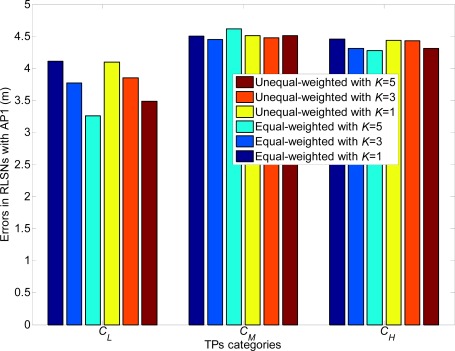
Relations of errors and deviations in equal and unequal-weighted RLSNs (*r* = 1 m).

From Figures 16–21, we can conclude that: (1) Although the error variations will become irregular under significantly large *r* conditions (e.g., *r* = 4 m), the errors will generally decrease as *K* increases and *r* decreases; (2) The errors are more sensitive to the *σ* values compared to the values of *K*; (3) The errors in equal-weighted RLSNs are slightly smaller compared to the unequal-weighted ones, which is also accordance with our previous analytical and numerical results.

The relations of the localization errors and standard deviations in equal and unequal RLSNs are also presented in Figure 21. Further, there are three categories of TPs to be considered: *C_L_*, *C_M_* and *C_H_*. The categories *C_L_* and *C_H_* consist of the front and last 11 TPs respectively with the largest and smallest standard deviations from AP1, while the other 11 TPs are included in *C_M_*. The errors are more sensitive to the variations of *σ*, especially from categories *C_L_* to *C_M_*. Generally, larger localization errors will result as the standard deviation always increases as expected.

## 5. Conclusions and Challenges

This paper has offered a preliminary analysis of the linear expected errors in the equal and unequal-weighted RLSNs with in-building Wi-Fi Gaussian linear fingerprints, and also introduced the mathematical relations of the linear expected errors (*ε_K_* or *ε_WK_*), number of neighbors (*K*), number and interval of calibrated RPs (*N*_RP_ and *r*) and standard deviations of the new sensed RSS at TP *σ*. The objective of this paper is that the suggested relations can be employed for a better design of the high-accurate, low-cost and real-time fingerprint-based in-building RADAR localization system in RLSNs, either through the judicious recording of the fingerprints, or through the optimal deployment of the system architectures and devices.

From the mathematical relations, numerical and experimental results proposed in this paper, there three observations can be made as follows: (1) In the equal and unequal-weighted RLSNs, the degree of influence on the linear expected errors is *r* > *σ* > *K* with a given value of *N*_RP_; (2) The error performance of equal-weighted RLSNs slightly outperforms the unequal-weighted ones in logarithmic Gaussian attenuation channels; (3) The expected error has great linear dependence on the values of *N*_RP_.

However, the following three challenges should form parts of our ongoing work: (1) Because the ideal logarithmic Gaussian attenuation channel utilized in this paper cannot be always satisfied or approximated in real in-building linear environments (e.g., the straight corridors), much attention will also be paid to some other typical models, like the Rayleigh and Rice distributions in the logarithmic channel with break point(s); (2) If the RPs are not calibrated on one side of the AP, the mathematical relations about the linear expected errors addressed in this paper will be changed because the test point will no longer satisfy the uniform distributions in the target location areas (e.g., the probability of the TP’s positions belonging to the range of (0, *d*_1_) will be doubled); (3) If there are three or more APs to be considered, for the WKNN localization algorithm, the confidence probability of each neighbor to be selected as the most RSS-adjacent RP should be calculated by a joint probability integral.

## Figures and Tables

**Table 1.** Notations and parameters.

**Items**	**Description**
*N*_RP_	Number of RPs.
*R_i_*(*i* = 1, ⋯ ,*N*_RP_)	Physical position of the *i*-th RP.
*d_i_ = ir*(*i* = 1, ⋯ ,*N*_RP_)	Physical distance between the *i*-th RP and AP (in meter).
*d_T_*	Physical distance between the TP and AP (in meter).
*r*	Interval of distance-adjacent RPs (in meter).
*δ*	Physical distance between the *i*-th RP and TP (in meter).
*P_t_*	Transmit power of AP (in dBm).
*P_i_*(*i* = 1, ⋯ ,*N*_RP_)	Pre-sensed RSS-mean at *R_i_* (in dBm).
$\bar{P_{T}}$	Expectation of the new sensed RSS at TP in Gaussian distribution (in dBm).
*σ*	Standard deviation of the new sensed RSS at TP (in dBm).
*L*_0_	Path loss in the first meter in logarithmic attenuation channel (in dBm).
*α*	Path loss exponent in logarithmic attenuation channel.
*E_x_*{*f*(*x*)}(*x* = *i,j,δ*)	Expectation of the function *f*(*x*) with respect to the variable *x*.
$C_{\text{KNN}}^{*}$	Estimated position by the KNN localization algorithm.
$C_{\text{WKNN}}^{*}$	Estimated position by the WKNN localization algorithm.
